# Supplementary figures and images for: Pathogenicity, Host Resistance, and Genetic Diversity of Fusarium Species under Controlled Conditions from Soybean in Canada
Source: J Fungi (Basel). 2024 Apr 23;10(5):303. doi: 10.3390/jof10050303 (PMC11122035; doi:10.3390/jof10050303)

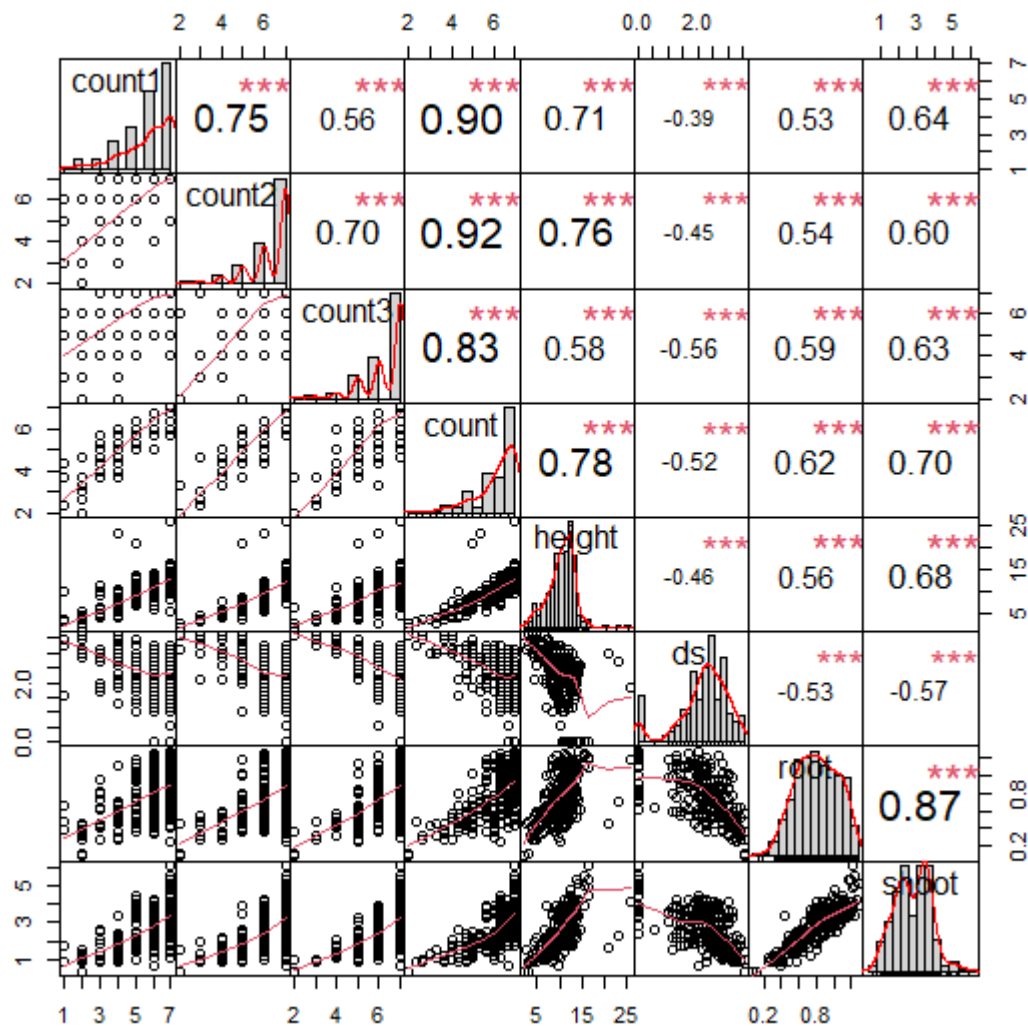

Supplement: Supplementary file 1 [file jof-10-00303-s001.zip › Supplementary Figure S1_Correlation analysis of all traits in pathogenictiy test under controlled conditions.pdf]

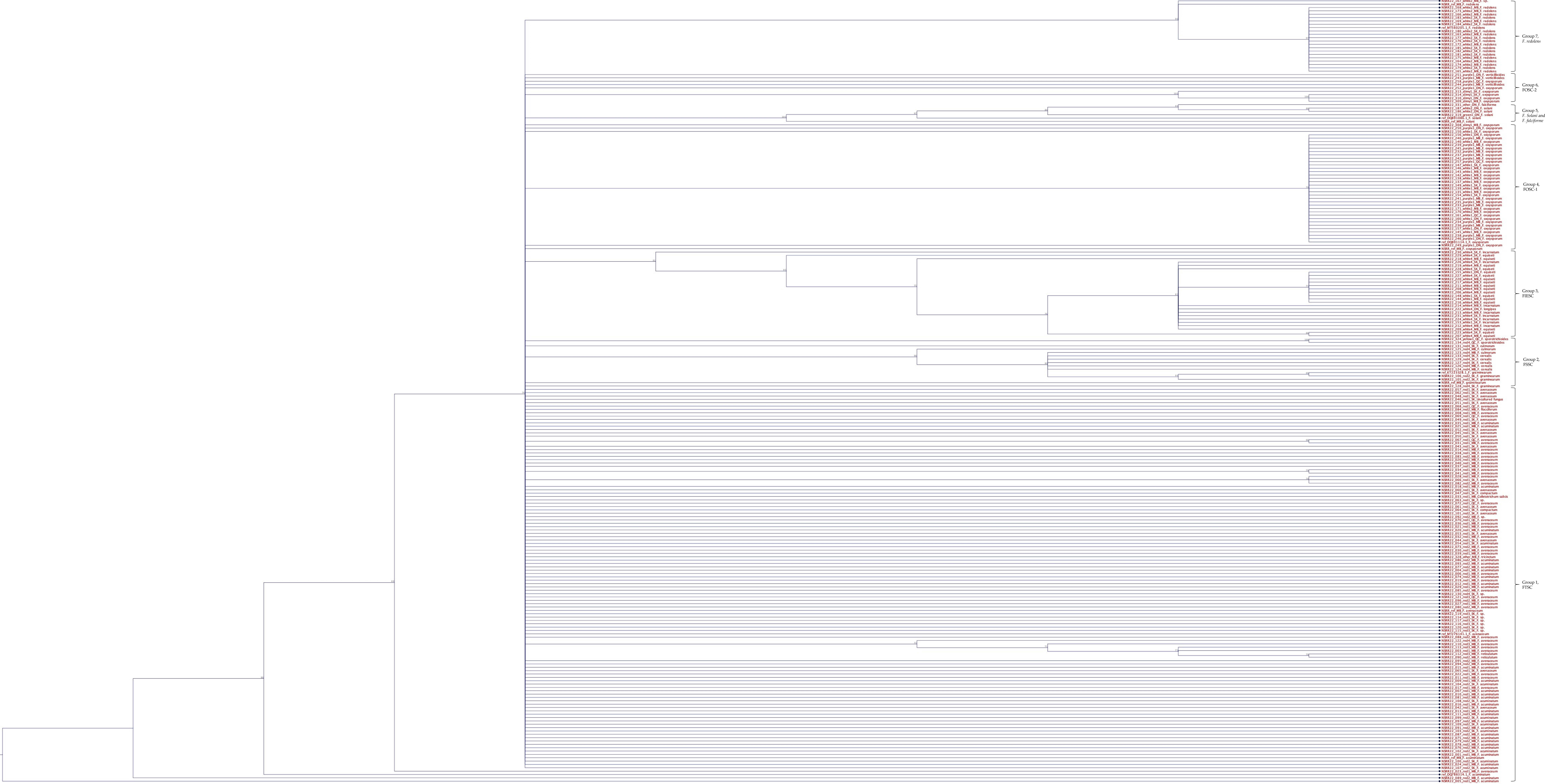

Supplement: Supplementary file 1 [file jof-10-00303-s001.zip › Supplementary Figure S2_phylogenetic tree of 221 isolates identified as Fusarium spp by ITS sequence.pdf]

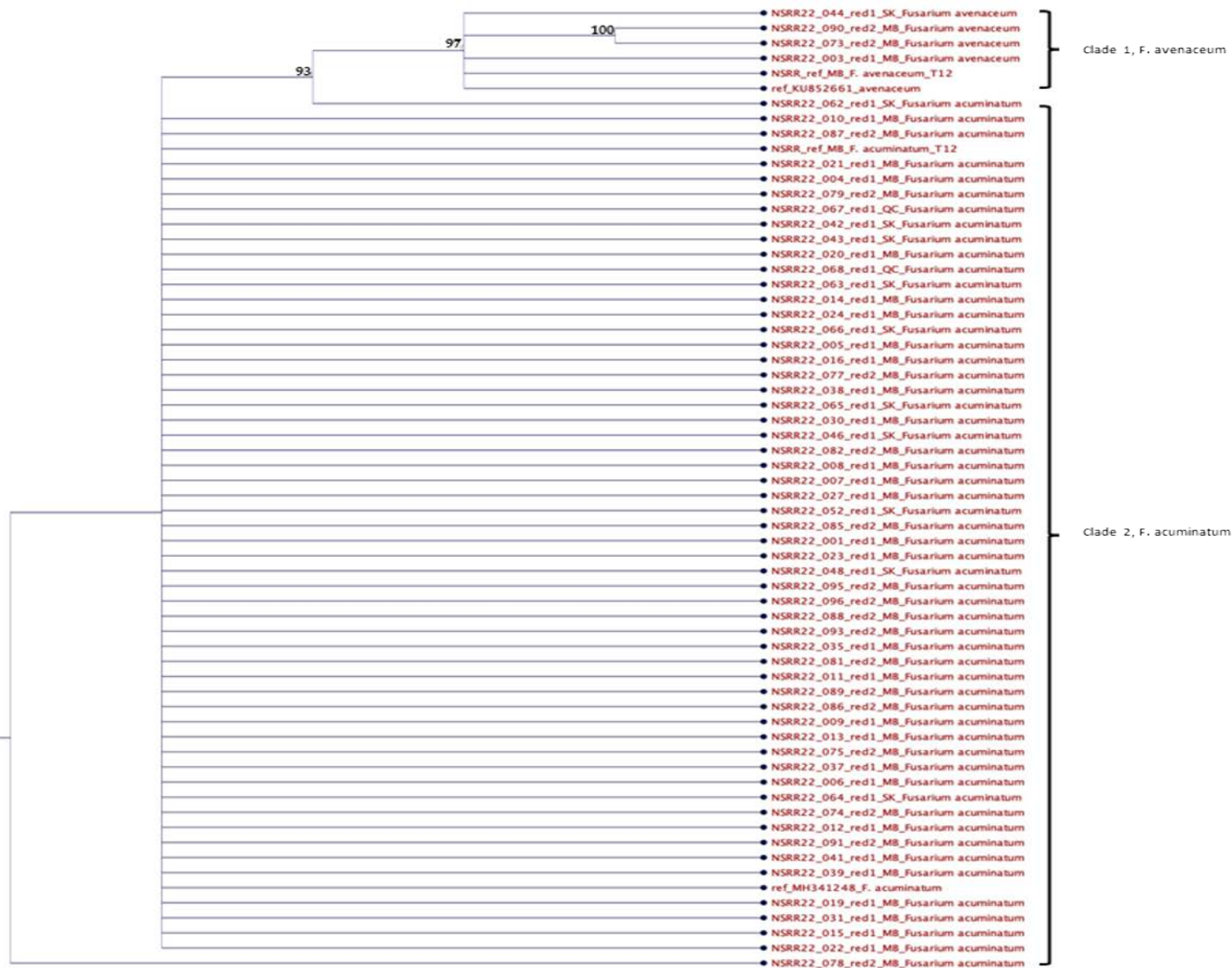

Supplement: Supplementary file 1 [file jof-10-00303-s001.zip › Supplementary Figure S3__phylogenetic tree identified as Fusarium spp by T12 sequence.pdf]
